# Corylin inhibits LPS-induced inflammatory response and attenuates the activation of NLRP3 inflammasome in microglia

**DOI:** 10.1186/s12906-018-2287-5

**Published:** 2018-08-15

**Authors:** Ming-Yii Huang, Chia-En Tu, Shu-Chi Wang, Yung-Li Hung, Chia-Cheng Su, Shih-Hua Fang, Chi-Shuo Chen, Po-Len Liu, Wei-Chung Cheng, Yu-Wei Huang, Chia-Yang Li

**Affiliations:** 1 Department of Radiation Oncology, Cancer Center, Kaohsiung Medical University Hospital, Kaohsiung Medical University, Kaohsiung, 80708 Taiwan; 2 Graduate Institute of Medicine, College of Medicine, Kaohsiung Medical University, Kaohsiung, 80708 Taiwan; 3 Department of Medical Laboratory Science and Biotechnology, Kaohsiung Medical University, Kaohsiung, 80708 Taiwan; 4 Institute of Health and Sports Science & Medicine, Juntendo University, Chiba, 270-1695 Japan; 5 Division of Urology, Department of Surgery, Chi-Mei Medical Center, Tainan, 71004 Taiwan; 6 Department of Senior Citizen Service Management, Chia Nan University of Pharmacy and Science, Tainan, 71710 Taiwan; 7 Institute of Athletics, National Taiwan University of Sport, Taichung, 40404 Taiwan; 8 Department of Biomedical Engineering and Environmental Sciences, National Tsing Hua University, Hsinchu, 30013 Taiwan; 9 Department of Respiratory Therapy, College of Medicine, Kaohsiung Medical University, Kaohsiung, 80708 Taiwan; 10 Graduate Institute of Biomedical Sciences, Research Center for Tumor Medical Science, and Drug Development Center, China Medical University, Taichung, 40402 Taiwan; 11 Department of Radiation Oncology, Kaohsiung Chang Gung Memorial Hospital, Kaohsiung, 83301 Taiwan

**Keywords:** Microglia, Corylin, MAPK signaling pathway, NLRP3 inflammasome, Anti-inflammation

## Abstract

**Background:**

Inflammation has been found to be associated with many neurodegenerative diseases, including Parkinson’s and dementia. Attenuation of microglia-induced inflammation is a strategy that impedes the progression of neurodegenerative diseases.

**Methods:**

We used lipopolysaccharide (LPS) to simulate murine microglia cells (BV2 cells) as an experimental model to mimic the inflammatory environment in the brain. In addition, we examined the anti-inflammatory ability of corylin, a main compound isolated from *Psoralea corylifolia* L. that is commonly used in Chinese herbal medicine. The production of nitric oxide (NO) by LPS-activated BV2 cells was measured using Griess reaction. The secretion of proinflammatory cytokines including tumor necrosis factor (TNF-α), interleukin-1β (IL-1β) and interleukin-6 (IL-6) by LPS-activated BV2 cells was analyzed using enzyme-linked immunosorbent assay (ELISA). The expression of inducible NO synthase (iNOS), cyclooxygenase-2 (COX-2), nucleotide-binding oligomerization domain-like receptor containing pyrin domain 3 (NLRP3), apoptosis-associated speck-like protein containing a caspase-activation and recruitment domain (ASC), caspase-1, IL-1β and mitogen-activated protein kinases (MAPKs) in LPS-activated BV2 cells was examined by Western blot.

**Results:**

Our experimental results demonstrated that corylin suppressed the production of NO and proinflammatory cytokines by LPS-activated BV2 cells. In addition, corylin inhibited the expression of iNOS and COX-2, attenuated the phosphorylation of ERK, JNK and p38, decreased the expression of NLRP3 and ASC, and repressed the activation of caspase-1 and IL-1β by LPS-activated BV2 cells.

**Conclusion:**

Our results indicate the anti-inflammatory effects of corylin acted through attenuating LPS-induced inflammation and inhibiting the activation of NLRP3 inflammasome in LPS-activated BV2 cells. These results suggest that corylin might have potential in treating brain inflammation and attenuating the progression of neurodegeneration diseases.

## Background

Inflammation has been found to be associated with pathogenesis of various neurodegenerative diseases such as Alzheimer’s disease and Parkinson’s disease [1]. Microglia, which are the resident macrophages in the central nervous system (CNS), play a critical role in regulating immune response and neuronal homeostasis [2, 3]. Upon the introduction of stimuli such as pathogens, inflammation and brain injury, microglia become rapidly activated and initiate an inflammatory cascade in response to exogenous or endogenous stimuli such as pathogens, inflammation and brain injury [4]. The activated microglia secrete several inflammatory cytokines and neurotoxic mediators such as tumor necrosis factor-α (TNF-α), interleukin-1β (IL-1β), IL-6 and nitric oxide (NO), while augmenting neurodegeneration and neuronal death [5–7]. The germline-encoded pattern recognition receptors and Toll-like receptor 4 (TLR4) are not only a major receptor in detection of bacterial component lipopolysaccharides (LPS), but also recognize heat-shock proteins and other danger-associated molecular patterns from damaged cells. TLR4 mediates several inflammatory signaling pathways and produces pro-inflammatory cytokines and mediators in response to infection, injury and toxin. Especially, TLR4 recognizes extracellular deposits of insoluble amyloid-β, which is a pivotal contributor to Alzheimer’s disease, and activates microglia [8]. Therefore, TLR4 activation critically contributes to neurodegeneration and neuronal death [9, 10], and is involved in development of neurodegenerative diseases [11, 12].

In TLR4-mediated inflammatory signaling pathways, mitogen-activated protein kinases (MAPKs) crucially regulate the production of pro-inflammatory cytokines and mediators [13]. The activated MAPKs trigger downstream transcription factors such as activator protein 1 and nuclear factor-κB (NF-κB), and produce pro-inflammatory cytokines and mediators [14]. Additionally, inducible NO synthase (iNOS) and cyclooxygenase-2 (COX-2) are produced and synthesize neurotoxic mediators NO and prostaglandin E_2_ (PGE_2_) by TLR4-mediated inflammatory signaling respectively [15]. Therefore, targeting the TLR4 signaling pathway is considered a method of improving neuroinflammation-related diseases [16].

On the other hand, the nucleotide-binding oligomerization domain-like receptor containing pyrin domain 3 (NLRP3) inflammasome is an essential regulator to produce IL-1β and is considered to regulate the progression of several neurodegenerative diseases [17]. The bacterial LPS or amyloid-β induce TLR4 signaling transduction pathway, mediate NF-κB activity, and produce NLRP3 and IL-1β precursors. NLRP3, an apoptosis-associated speck-like protein containing a caspase-activation and recruitment domain (ASC), and pro-caspase-1 combine to form an NLRP3 inflammasome complex [18]. Additionally, necrotic cells release ATP and trigger P2X7 receptor signaling, and then pro-caspase-1 is converted into activated caspase-1. The activated caspase-1 cleaves IL-1β precursors and converts these into mature IL-1β [19]. The secretion of IL-1β augments inflammation and neurotoxicity, while leading to neurodegeneration and neuronal death. Thus, the inhibition of NLRP3 inflammasome is considered a therapeutic target of neurodegenerative diseases [17, 19, 20].

Nonsteroidal anti-inflammatory drugs (NSAIDs) have been demonstrated to exhibit neuroprotective effects, but long-term NSAID treatment might induce side effects [4]. Flavonoids are abundantly present in plant, fruits and vegetables, and have been indicated to exert several types of anti-inflammatory effects [21, 22]. *Psoralea corylifolia* L. has been widely used as a kidney tonifying herbal medicine for treating many diseases such as osteoporosis [23], leucoderma and inflammatory diseases of the skin in Asian countries [24]. Corylin is a main flavonoid that is isolated from *Psoralea corylifolia* L. Previous studies indicated that corylin has various pharmaceutical effects, including anti-cancer [25, 26] and anti-inflammatory properties [27, 28]. However, the anti-inflammatory effects of corylin on microglia remain unclear.

In this study, we investigated the effects of corylin on LPS-induced inflammation by murine brain microglia, BV2 cells. Firstly, we evaluated the effects of corylin on the production of pro-inflammatory cytokines (TNF-α, IL-6 and IL-1β) and a neurotoxic mediator (NO) by LPS-activated BV2 cells. Secondly, we examined the effects of corylin on the expression of iNOS, COX-2 and MAPKs by LPS-activated BV2 cells. Finally, we tested the effect of corylin on the activation of NLRP3 inflammasome by LPS-activated BV2 cells.

## Methods

### Reagents

DMEM, penicillin, and streptomycin purchased from Gibco-BRL (Life Technologies, Grand Island, NY, USA). Fetal bovine serum (FBS) was purchased from Hyclone Laboratories (Logan, UT, USA). LPS (from *E. coli* 0111:B4), Griess reagent, 3-(4, 5-dimethylthiazol-2-yl)-2, 5-diphenyl tetrazolium bromide (MTT), RIPA buffer, protease inhibitors, and phosphatase inhibitors were purchased from Sigma Aldrich (St. Louis, MO, USA). TNF-α, IL-6 and IL-1β ELISA kits were purchased from eBioscience (San Diego, CA, USA). BCA protein assay kit, ECL chemiluminescence substrate, and Hoechst 33,342 were obtained from Thermo Scientific (Waltham, MA, USA). Rabbit antibodies against mouse iNOS, COX-2, ASC, IL-1β, β-actin and secondary antibodies were obtained from Santa Cruz Biotechnology (Santa Cruz, CA, USA). Rabbit antibodies against mouse phospho-JNK, JNK, phospho-p38 MAPK, p38 MAPK, phospho-ERK, ERK, NLRP3 and caspase-1 (p20) were purchased from Cell Signaling (Farmingdale, NY, USA). Corylin (purity > 98%) was obtained from ChemFaces (Wuhan, Hubei, China) and analyzed by the Limulus amebocyte lysate assay (Associates of Cape Cod, Falmouth, MA, USA) to avoid the possibility of endotoxin contamination. Results indicated that corylin had undetectable level of endotoxin (< 0.03 EU/mL, data not shown). For the treatment of corylin, corylin was dissolved in DMSO at a stock concentration of 50 mM, then further diluted in the culture medium at a final DMSO concentration of ≤0.02%.

### Cell culture

Murine BV2 microglial cells were purchased from the Food Industry Research and Development Institute (Hsinchu, Taiwan) and cultured in DMEM supplemented with 10% FBS and antibiotics (100 U/mL penicillin and 100 U/mL streptomycin) in a humidified atmosphere of 5% CO_2_ at 37 °C and passaged every 2–3 days to maintain growth.

### NO assay

The Griess assay measures the level of accumulated nitrite (NO_2_^−^), a metabolite of NO, in culture supernatant by the Griess reagent. BV2 cells were seeded in a 96-well plate at a density of 1 × 10^5^ cells per well and incubated overnight. Cells were pre-treated with various concentrations of corylin (0 to 10 μM) for 1 h, and then were treated with LPS (1 μg/mL) for 24 h. The supernatant of cell culture was collected and the concentration of NO was measured by the Griess reagent.

### MTT assay

BV2 cells were seeded in a 96-well plate at a density of 1 × 10^5^ cells per well and incubated overnight. Cells were pre-treated with various concentrations of the corylin (0 to 10 μM) for 1 h, and then treated with LPS 1 μg/mL for 24 h. Cell viability was assayed by MTT assay following the manufacturer’s instructions (Sigma, St. Louis, MO, USA). Cell viability was calculated using the equation: (mean OD of treated cells/mean OD of control cells) × 100.

### Enzyme-linked Immunosorbent assay (ELISA)

BV2 cells were seeded in 96-well plate at a density of 1 × 10^5^ cells per well and incubated overnight. Cells were pre-treated with different concentrations (0 to 10 μM) of corylin for 1 h, and then treated with LPS 1 μg/mL for 24 h. The supernatant of cell culture was collected and analyzed by ELISA according to the manufacturer’s protocol (eBioscience, San Diego, CA, USA).

### Western blotting

Cells were lysed by RIPA buffer with protease inhibitors and phosphatase inhibitors and the concentration of protein was evaluated using the BCA protein assay reagent following the manufacturer’s instructions (Thermo Scientific, Waltham, MA, USA). Aliquots of equal amounts of proteins from the cells were subjected to SDS–PAGE. Thereafter, proteins were electrophoretically transferred to PVDF membranes. The membranes were incubated with 5% skim milk to block nonspecific protein binding and incubated with primary antibodies at 4 °C overnight. After washing 3 times with Tris-buffered saline/Tween 20 (TBST), the blots were hybridized with horseradish peroxidase-conjugated secondary antibodies for 1 h at room temperature. Then, the blots were washed three times with TBST, and the specific immunoreactive protein bands were detected by ECL chemiluminescence substrate. The signals were captured and the band intensities were quantified using Bio-Rad ChemiDoc XRS^+^ system (Bio-Rad Laboratories, Inc., Hercules, CA, USA).

### Immunofluorescence staining

The formation of inflammasome was imaged by ASC/caspase-1 immunofluorescence staining. BV2 cells were seeded on 12-mm glass coverslips in 24 well-plates overnight. Cells were incubated with 10 μM corylin prior to LPS treatment (1 μg/mL) for 24 h. Then, cells were fixed with 4% paraformaldehyde and permeabilized using 0.2% Triton X-100 in PBS, and cells were incubated with anti-ASC and anti-caspase-1 primary antibodies overnight. Then, cells were washed with PBS to remove the excessive primary antibodies, and incubated with fluorescent secondary antibodies. The cell nucleus was labeled with Hoechst 33,342. High magnification fluorescent images were taken using an inverted epi-fluorescent microscope (Nikon-Ti, Nikon, Japan) with 60× oil immersion objectives. The localization and expression of ASC and caspase-1 were processed using NIS-Elements software (Nikon, Japan) and ImageJ software (National Institutes of Health, Bethesda, MD, USA).

### Statistical analysis

All experiments were performed at least in triplicate, with data presented as mean ± standard deviation (SD) of independent experiments and analyzed using IBM SPSS Statistics v.19 (IBM Corp., Armonk, NY, USA). Comparisons between control and treatment groups were made using Student’s *t*-test. The significant difference was set at *: *p* < 0.05; **: *p* < 0.01; ***: *p* < 0.001.

## Results

### Corylin inhibits the production of NO and the expression of iNOS and COX-2 in LPS-activated murine microglial cells

**Fig. 1:**
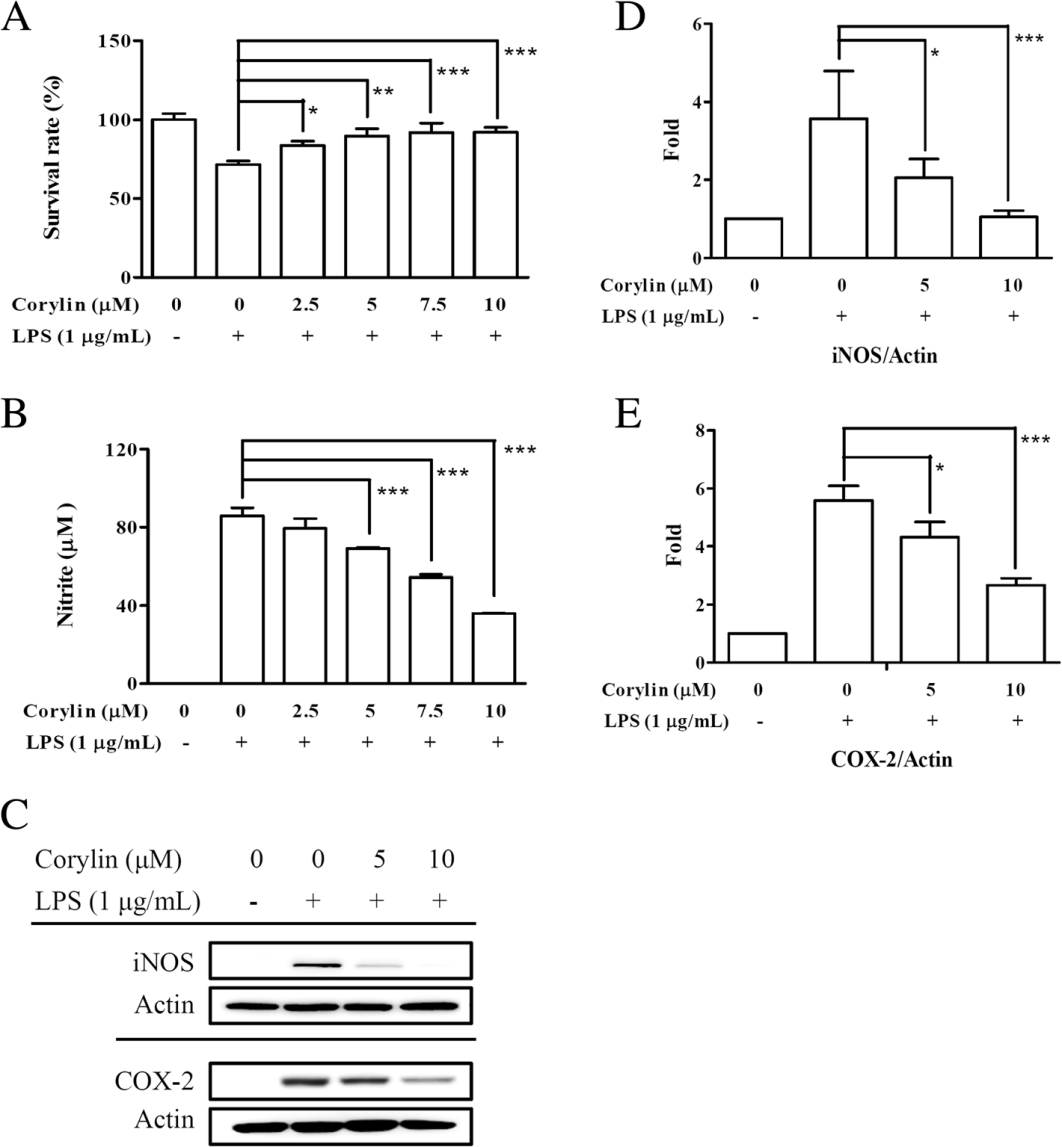
Effects of corylin on the cell viability and the production of NO by LPS-activated BV2 cells. BV2 cells were pre-treated with various doses of corylin for 1 h, and then treated with LPS (1 μg/mL) for 24 h. **a** The survival rate of BV2 cells was measured by MTT assay. **b** The production of NO in the cell culture supernatant was detected by Griess reaction. **c** The expression of COX-2 and iNOS was examined by Western blot. Blots are representative blots. The relative ratio of (**d**) iNOS /β-actin and (**e**) COX-2/β-actin are shown. Data represent mean ± SD of three independent experiments (*: *p* < 0.05; **: *p* < 0.01; ***: *p* < 0.001 vs. LPS alone)

To avoid the toxic effects of corylin, we performed MTT assay to examine the cell survival after corylin and LPS treatments. BV2 cells were pre-treated with corylin at different doses (0 to 10 μM) for 1 h, and then treated with LPS 1 μg/mL for 24 h. As shown in Fig. 1a, there was no toxic effect of corylin when BV2 cells were treated with corylin 0 to 10 μM. In addition, corylin protected BV2 cells against LPS-induced cell death (Fig. 1a). Both iNOS and COX-2 are critical inflammation-related enzymes involved in producing nitric oxide and prostaglandins respectively [15]. To examine the effect of corylin on LPS-induced NO production, BV2 cells were pre-treated with various doses of corylin (0 to 10 μM) for 1 h, and then stimulated with LPS (1 μg/mL) for 24 h. The production of NO was analyzed by Griess reagent assay. Our experimental results showed that corylin significantly suppressed the production of NO by LPS-stimulated BV2 cells in a dose-dependent manner (Fig. 1b). We further examined the effect of corylin on the expression of iNOS and COX-2 using Western blot. Our results indicated that corylin suppressed the expression of iNOS and COX-2 by LPS-stimulated BV2 cells, as compared with LPS alone (Fig. 1c, d and e).

### Corylin represses LPS-induced production of pro-inflammatory cytokines in murine microglial cells

**Fig. 2:**
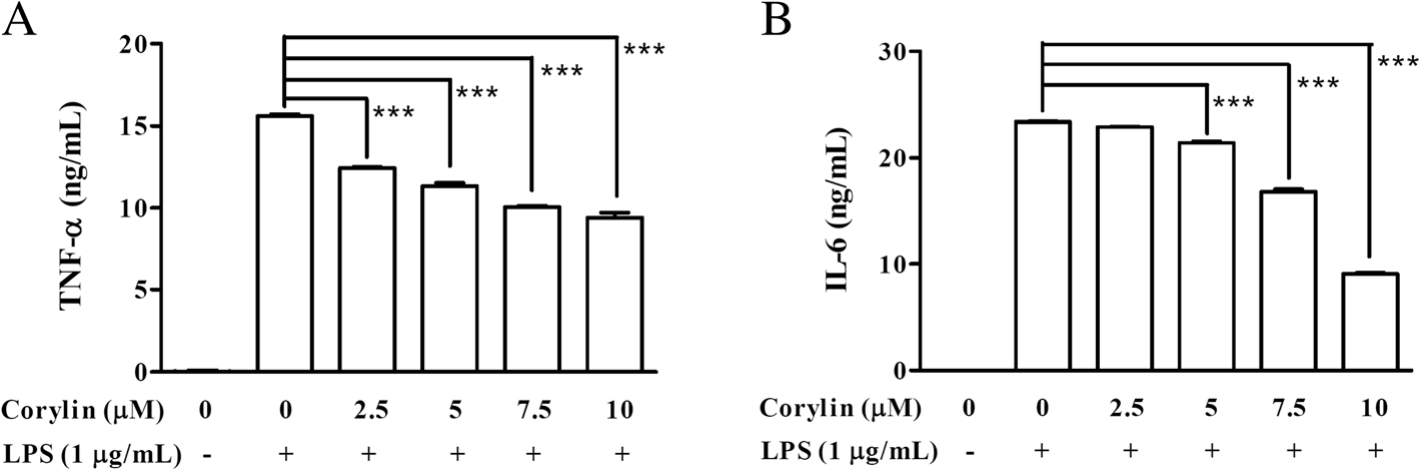
Effects of corylin on the production of proinflammatory cytokines by LPS-activated BV2 cells. BV2 cells were pre-treated with various doses of corylin for 1 h, and then treated with LPS (1 μg/mL) for 24 h. The expressions of **a** TNF-α and **b** IL-6 in the cell culture supernatant were analyzed using ELISA. Data represent mean ± SD of three independent experiments (*: *p* < 0.05; **: *p* < 0.01; ***: *p* < 0.001 vs. LPS alone)

Since TNF-α and IL-6 are critical pro-inflammatory cytokines in response to LPS, we further tested whether corylin affected the production of pro-inflammatory cytokines induced by LPS in murine microglial cells. BV2 cells were pre-treated with various concentrations of corylin for 1 h, and then treated with LPS (1 μg/mL) for 24 h. The production of TNF-α and IL-6 was determined by ELISA. As shown in Fig. 2, corylin inhibited both TNF-α and IL-6 production by LPS-activated BV2 cells in a concentration-dependent manner.

### Corylin suppresses the phosphorylation of MAPKs by LPS-activated murine microglial cells

**Fig. 3:**
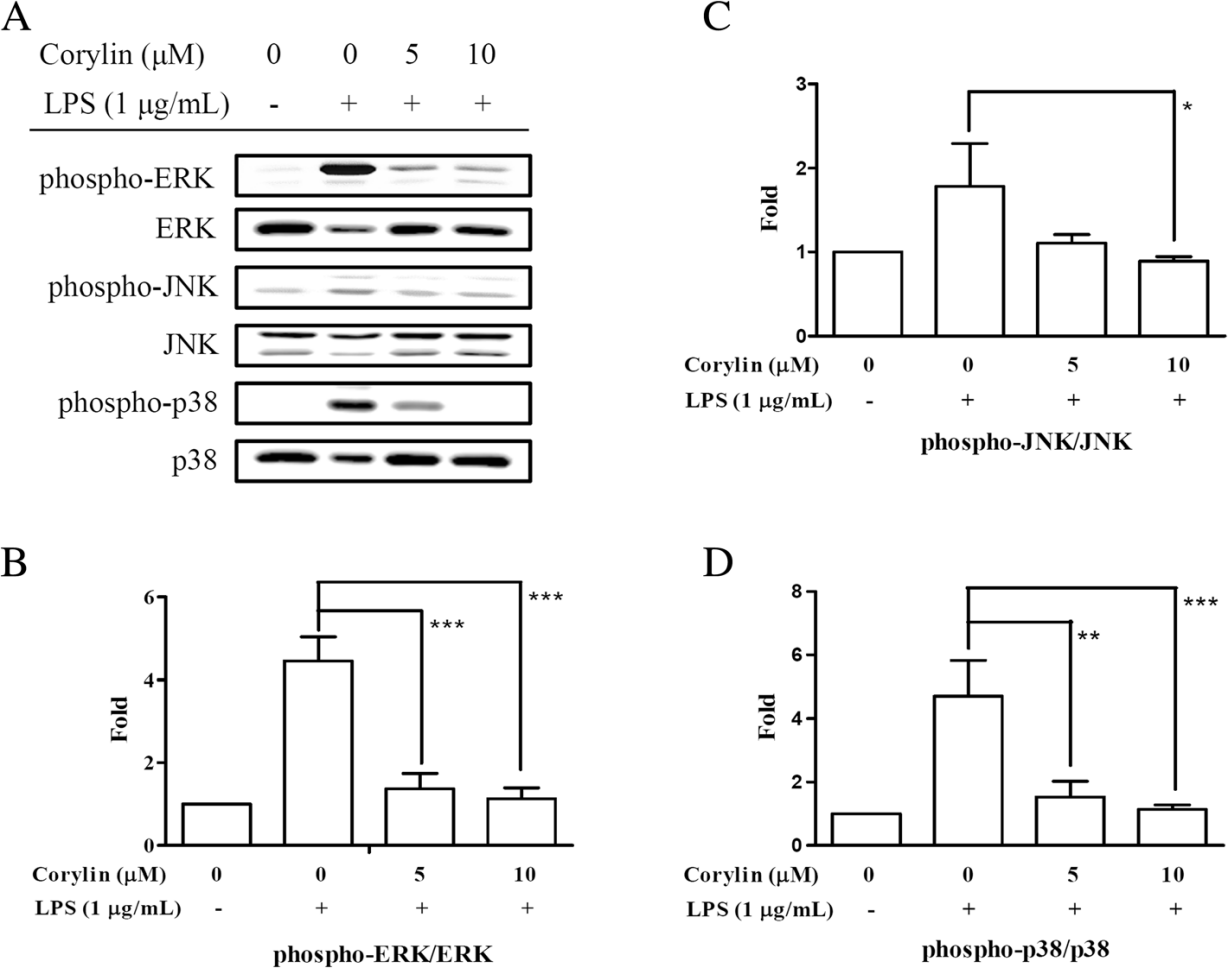
Effects of corylin on MAPK signaling pathway by LPS-activated BV2 cells. Cells were incubated with various doses of corylin for 1 h, and then treated with LPS (1 μg/mL) for 24 h. **a** Western blot analysis of phospho-JNK, JNK, phospho-p38 MAPK, p38 MAPK, phospho-ERK and ERK. Blots are representative blots. **b**, **c**, **d** Quantitation was performed on three independent experiments and presented as the means ± SD. The relative fold was phosphorylation to dephosphorylation ratio and compared to untreated samples (*: *p* < 0.05; **: *p* < 0.01; ***: *p* < 0.001 vs. LPS alone)

MAPKs (JNK, p38 MAPK and ERK) phosphorylate a wide range of substrate proteins including transcription factors, which play an important role in regulating inflammatory responses and modulating the production of pro-inflammatory mediators and cytokines [29]. To investigate the effect of corylin on the activation of MAPKs, BV2 cells were incubated with various doses of corylin for 1 h, and then treated with LPS (1 μg/mL) for 24 h. The expression levels of phospho-JNK, JNK, phospho-p38 MAPK, p38 MAPK, phospho-ERK and ERK were examined by Western blot. As shown in Fig. 3, the phosphorylation of JNK, p38 MAPK and ERK was markedly elevated after LPS stimulation. Treatment with corylin (5 and 10 μM) significantly decreased LPS-induced phosphorylation of JNK, p38 MAPK and ERK in BV2 cells (Fig. 3).

### Corylin suppresses the production of IL-1β through attenuating the activation of NLRP3 inflammasome in murine microglial cells

**Fig. 4:**
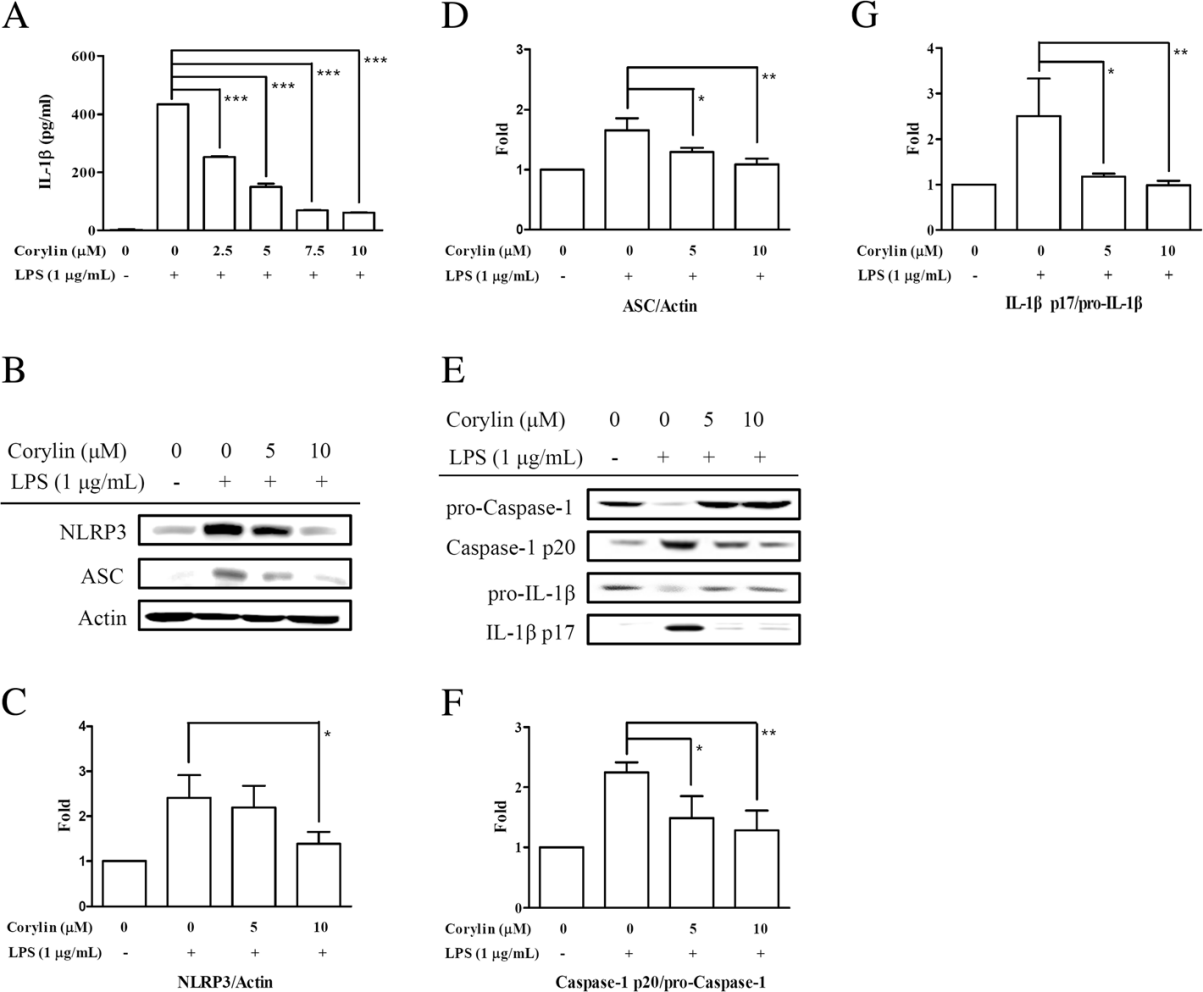
Effects of corylin on the activation of NLRP3 inflammasome by LPS-activated BV2 cells. BV2 cells were incubated with various doses of corylin for 1 h, and then treated with LPS (1 μg/mL) for 24 h or 48 h. **a** The level of IL-1β in the cell culture supernatant was examined by ELISA (***: *p* < 0.001 vs. LPS alone). **b** The expression levels of NLRP3 and ASC were examined by Western blot. Blots are representative blots. **c**, **d** Quantitation was performed on three independent experiments and presented as the means ± SD. The expression of β-actin was used as an internal control. The relative ratios of NLRP3/β-actin and ASC/β-actin are shown (*: *p* < 0.05; **: *p* < 0.01 vs. LPS alone). **e** The expression levels of pro-caspase-1, mature caspase-1 (p20), pro-IL-1β, and mature IL-1β (p17) were examined by Western blot. Blots are representative blots. Quantitation was performed on three independent experiments and presented as the means ± SD. The relative ratio of **f** mature caspase-1 (p20)/immature caspase-1 and **g** mature IL-1β (p17)/immature IL-1β are shown (*: *p* < 0.05; **: *p* < 0.01 vs. LPS alone)

**Fig. 5:**
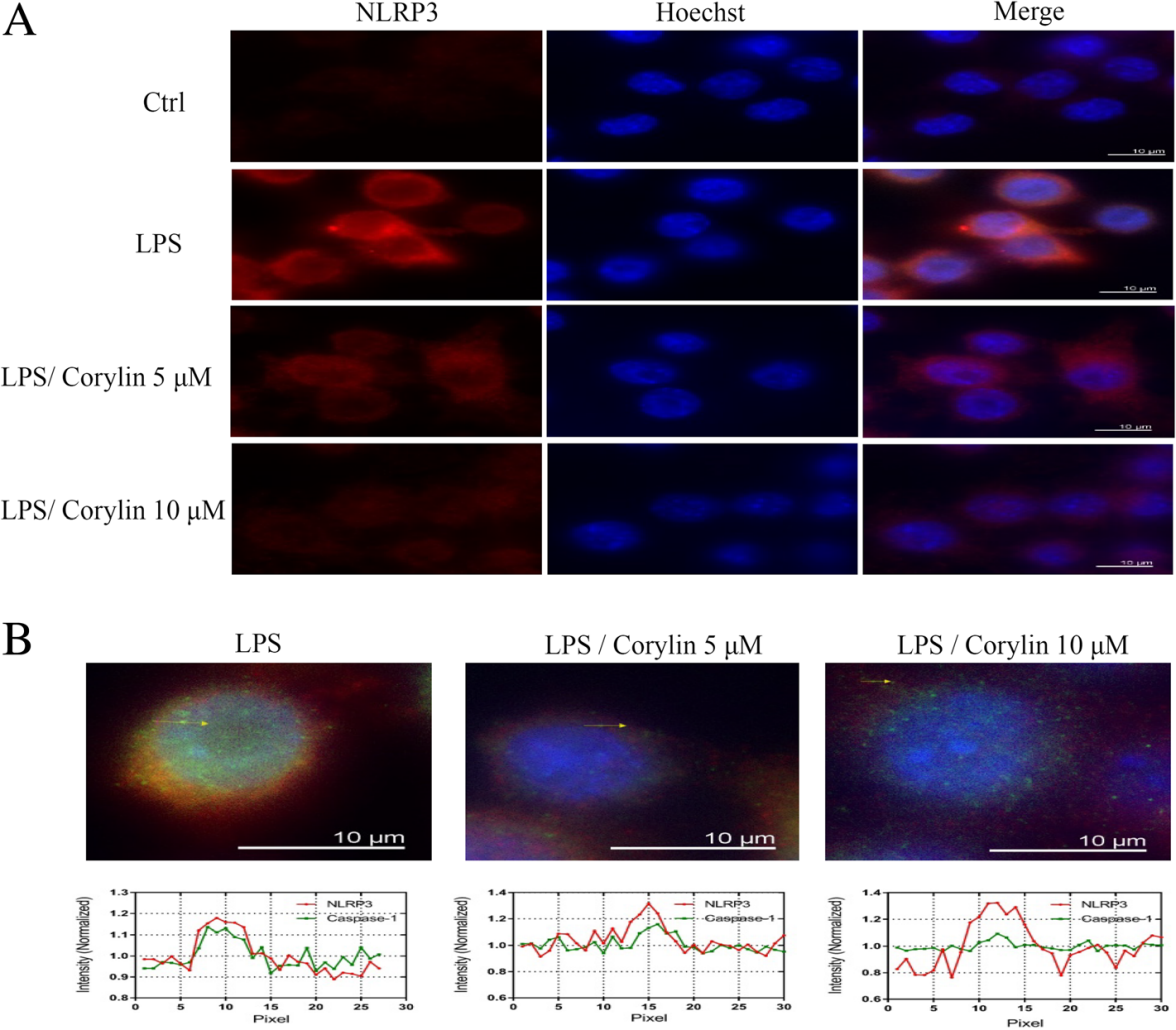
Effects of corylin on the expression of NLRP3 and colocalization of NLRP3 with caspase-1 (green) in LPS-activated BV2 cells. Cells were incubated with various doses of corylin for 1 h, and then treated with LPS (1 μg/mL) for 24 h. **a** Representative fluorescent microscopy images showing the expression of NLRP3 (red) in LPS-activated BV2 cells. The nuclei were stained with Hoechst 33,342 (blue). **b** Representative fluorescent microscopy images showing the colocalization of NLRP3 (red) with caspase-1 (green) in LPS-activated BV2 cells. The nuclei were stained with Hoechst 33,342 (blue). Scale bars, 10 μm

IL-1β is a critical proinflammatory cytokine, which subsequently enhances the production of both TNF-α and IL-6 [30] and promotes the generation of reactive oxygen species by microglia causing severe tissue and organ damage [20]. To investigate whether corylin affects the production of IL-1β by LPS-activated murine microglial cells, BV2 cells were pre-treated with various concentrations of corylin for 1 h, and then treated with LPS (1 μg/mL) for 48 h. The production of IL-1β was analyzed by ELISA. As shown in Fig. 4a, corylin suppressed the production of IL-1β by LPS-activated BV2 cells. The activation of NLRP3 inflammasome is an important innate immune pathway, which is critical for the production of active IL-1β, and is considered as a key contributor to the development of neuroinflammation [31]. We further examined whether corylin affects the activation of NLRP3 inflammasome in LPS-activated BV2 cells. Our results indicated that corylin reduced the expression of NLRP3 and ASC by LPS-activated BV2 cells (Fig. 4b, c and d). In addition, we also found that corylin decreased the expression of mature caspase-1 and mature IL-1β by LPS-activated BV2 cells (Fig. 4e, f and g). Moreover, we also confirmed that corylin inhibited the expression of NLRP3 using immunofluorescent staining (Fig. 5a) and demonstrated corylin inhibited the formation of inflammasome complex in LPS-activated BV2 cells (Fig. 5b).

## Discussion

Corylin, a main flavonoid isolated from *Psoralea corylifolia* L., has been demonstrated to exhibit various biological properties such as anti-tumor [25, 26] and anti-inflammatory effects [27, 28]; however, the effect of corylin on LPS-activated microglia has not been examined. To our knowledge, this is the first report indicating that corylin inhibits LPS-induced inflammatory response and attenuates the activation of NLRP3 inflammasome in murine brain microglia.

Activated microglia cause neuronal cell degeneration by secreting various pro-inflammatory cytokines and neurotoxic mediators [32]. In the present study, our results demonstrated that corylin inhibited the production of NO and suppressed the expression of iNOS and COX-2, meanwhile decreasing the secretion of TNF-α and IL-6 in LPS-activated murine microglial cells. As similar to our previous study, we found that corylin inhibits the expression of iNOS and COX-2 and decreases the production of NO and PGE_2_, and suppresses the secretion of TNF-α and IL-6 by LPS-activated macrophages [28]. Various research evidence indicates that higher concentrations of NO and COX-2 have neurotoxic effects and are associated with several neurodegeneration diseases [33–35]. In addition, a local release of proinflammatory cytokines (TNFα and IL6) causes the recruitment of leukocytes across the blood–brain barrier and amplifies the inflammatory reaction, consequently leading to neuro-inflammatory processes consequently [36]. Taken together, these results suggest that corylin could suppress the production of neurotoxic mediators (NO and COX-2) and pro-inflammatory cytokines (TNF-α and IL-6) during inflammation and might have benefits in attenuating neurotoxic effects.

MAPK cascade and its linked downstream transcription factor, NF-κB, play an important role in regulating the expression and production of several pro-inflammatory cytokines and mediators [14, 29]. In the present study, our results indicated that corylin significantly decreased LPS-induced phosphorylation of JNK, p38 MAPK and ERK by microglia. In addition, our previous study also demonstrated that corylin attenuates the phosphorylation of MAPKs by LPS-activated macrophages [28]. Moreover, corylin also suppresses the activation of NF-κB by LPS-activated macrophages [28]. Collectively, these results highlight that corylin inhibits LPS-induced pro-inflammatory cytokines and mediators through inhibition of MAPKs and NF-κ B signaling pathways in both macrophages and microglia.

NLRP3 inflammasome is the main regulator to produce IL-1β and is considered to regulate the progression of several neurodegenerative diseases [17, 37]. Our results demonstrated that corylin inhibits the production of IL-1β by LPS-activated microglia. In addition, we also found that corylin suppresses the expression of NLRP3, ASC, mature caspase-1 and mature IL-1β by LPS-activated microglia. These results demonstrate that corylin inhibits the production of IL-1β through attenuating the activation of the NLRP3 inflammasome.

## Conclusion

**Fig. 6:**
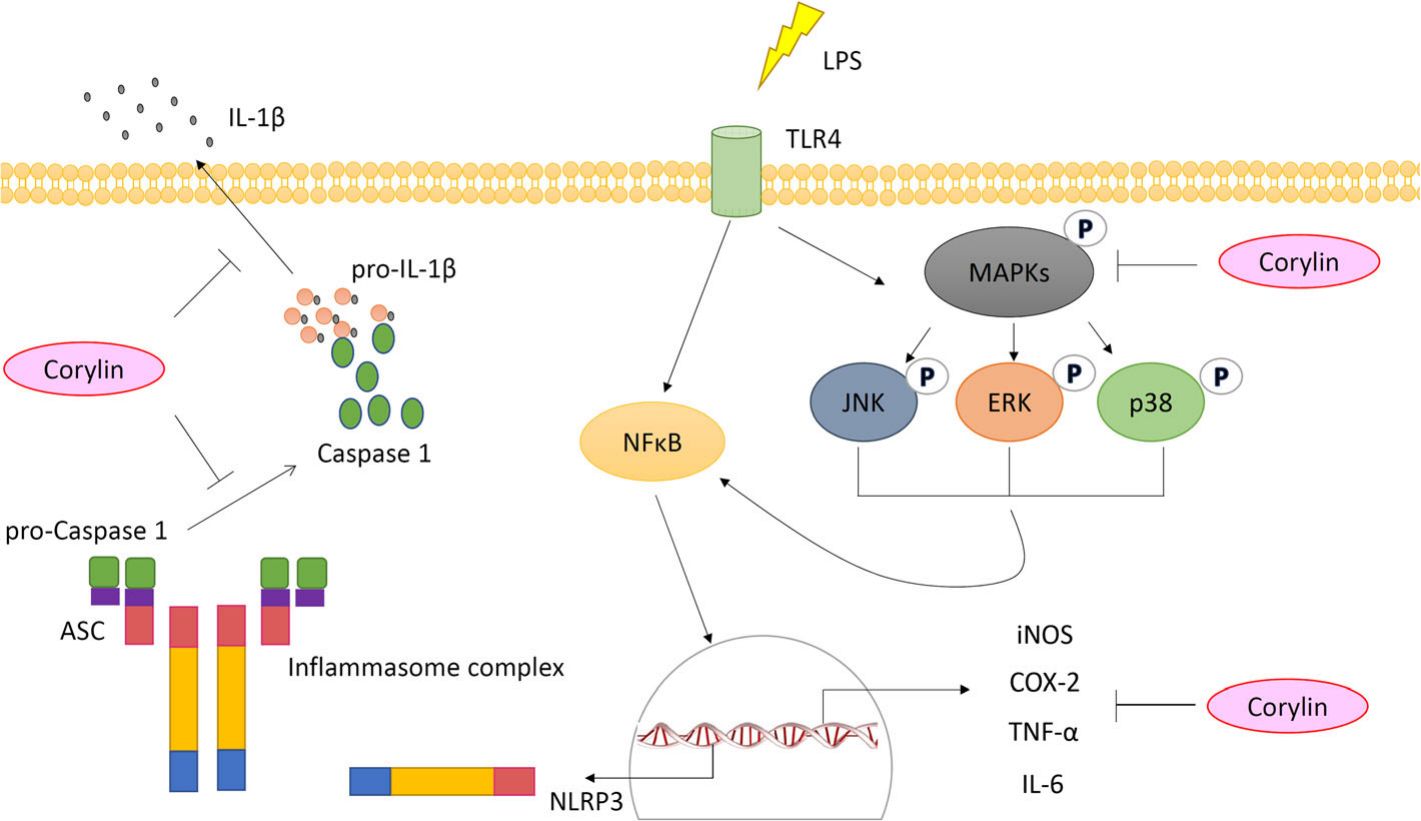
The potential mechanisms of corylin in suppression of LPS-induced inflammation and NLRP3 activation in microglia

The results of the present study showed that corylin inhibits LPS-induced inflammatory responses including decreasing the production of inflammatory mediators (NO), suppressing the expression of iNOS and COX-2, and inhibiting the secretion of proinflammatory cytokines (TNF-α, IL-6, and IL-1β) in murine microglial cells. In addition, corylin attenuated the activation of both MAPKs and NLRP3 inflammasome pathways in LPS-activated murine microglial cells (Fig. 6). Collectively, these results suggest that corylin has potential to inhibit neuroinflammation and might have significant benefits in treating neurodegeneration diseases.

## Abbreviations

ASC: Apoptosis-associated speck-like protein containing a caspase-activation and recruitment domain
COX-2: Cyclooxygenase-2
ELISA: Enzyme-linked immunosorbent assay
FBS: Fetal bovine serum
IL-1β: Interleukin-1β
IL-6: Interleukin-6
iNOS: Inducible NO synthase
LPS: Lipopolysaccharide
MAPKs: Mitogen-activated protein kinases
MTT: 3-(4, 5-dimethylthiazol- 2-yl)-2, 5-diphenyl tetrazolium bromide
NF-κB: Nuclear factor-κB
NLRP3: Nucleotide-binding oligomerization domain-like receptor containing pyrin domain 3
NO: Nitric oxide
NSAIDs: Nonsteroidal anti-inflammatory drugs
PGE2: Prostaglandin E2
SD: Standard deviation
TBST: Tris-buffered saline/Tween 20
TLR4: Toll-like receptor 4
TNF-α: Tumor necrosis factor
